# Essential Oils as Natural Defenders: Exploring Their Antioxidant and Antimicrobial Potential Against Multidrug‐Resistant (MDR) Bacteria

**DOI:** 10.1155/ijm/8834643

**Published:** 2026-05-03

**Authors:** Sahar Dahbi, Fatima-Ezzahra En-Naciri, Asmae Alaoui, Mouhcine Fadil, Yassine Ait Zengui, Zakaria Marmat, Fatimazahra Ouatiq, Mohammed Raouane, Souad Amghar

**Affiliations:** ^1^ Research Team “Lombricidae Improving Soil Productivity and Environment (LAPSE), Center Water, Natural Resources, Environment and Sustainable Development (CERNE-2D), Ecole Normale Supérieure de Rabat–Mohammed V University in Rabat, Rabat, Morocco; ^2^ Physio-Chemical Laboratory of Inorganic and Organic Materials (LPCMIO), Ecole Normale Supérieure de Rabat, Mohammed V University in Rabat, Rabat, Morocco, um5a.ac.ma; ^3^ Laboratory of Applied Organic Chemistry, Faculty of Sciences and Techniques, Sidi Mohamed Ben Abdellah University, Fez, Morocco, usmba.ac.ma

**Keywords:** antimicrobial activity, chemical composition, essential oils, multidrug resistant bacterium, therapeutic application

## Abstract

This study explores the potential of six essential oils (EOs): clove (*Syzygium aromaticum*); pennyroyal (*Mentha pulegium*); jasmine *(Jasmin officinal*); pin (*Pinus sylvestris* var. *sylvestris* L); rosemary (*Rosmarinus officinalis*); and peppermint (*Mentha x piperita*), extracted from aromatic and medicinal plants highlighting their bioactive properties. Phytochemical analysis was conducted throughout GC‐MS. The antioxidant activity was evaluated by DPPH method, and their antimicrobial potential was tested against three reference bacterial strains and one isolated multidrug‐resistant bacterium (*S. capitis*), using the tests of disc‐diffusion, minimum inhibitory, and bactericidal concentrations. The chemical composition identified major compounds including: eugenol 74.5% in *S. aromaticum*, pulegone 73.07% in *M. pulegium*, n‐hexyl cinnamaldehyde 23.61% in *J. officinal*, terpinen‐4‐ol 24.24% in *P. sylvestris*, eucalyptol 41.82% in *R. officinalis*, and menthol 44.67%, in *M. piperita*. In addition, *M. pulegium* showed the weakest antioxidant potential, whereas *S. aromaticum* was the most effective EO, as it illustrated the highest antioxidant effect and the strongest antimicrobial activity. In addition, its combination with each tested antibiotic significantly enhanced the antimicrobial activity against *S. capitis*, highlighting the synergistic potential of these combinations. This study fills a critical gap in the literature, as no prior research has examined these interactions. The statistical analysis revealed distinct groupings among the six EOs based on their chemical composition and biological activities. In this way, strong positive correlations were observed between major compounds, antibacterial, and antioxidant activities. This research highlights the promising use of some EOs as remarkable antioxidant and to combat infections caused by antibiotic‐resistant bacteria, indicating their promising therapeutic applications.

## 1. Introduction

*Staphylococcus capitis*, *Staphylococcus aureus*, *Escherichia coli*, and *Pseudomonas aeruginosa* are the leading causes of several diseases from minor skin infections, urinary tract, and gastrointestinal disorders to deadly contagions including pneumonia and bloodstream infections [1, 2]. Indeed, the recent classification of multidrug resistance created by the WHO arranged bacteria based on the urgent need for new antibiotics [3]. This classification placed *P. aeruginosa* and *E. coli* within the critical group of multidrug resistance. Meanwhile, *S. aureus* falls into the high‐priority group [3]. In this context, the use of EOs extracted from various medicinal and aromatic plants as novel agents against drug‐resistant microorganisms is a noteworthy avenue of investigation. They are considered an important source of compounds with various biological activities. Recent research attributed the antimicrobial potential of EOs not only to their major components but also to the synergistic or antagonistic effect of minor elements [4].

Therefore, the present study is aimed at determining the chemical composition, as well as the antioxidant activity of six essential oils (EOs) and shedding light on their potential as effective antimicrobial agents. Additionally, this study focuses on combining resistant antibiotics with selected EOs in order to assess their antimicrobial properties on an isolated multiresistant strain, *S. capitis*, suggesting their potential use in the food, cosmeceutical, and pharmaceutical industries. As far as we are aware, there have been no previous investigations into these particular interactions. Therefore, our research intends to address this knowledge gap.

These findings could offer important insights into the use of natural products for combating microbial infections. This research could also lay the groundwork for more research in developing new treatments to address antibiotic resistance.

## 2. Materials and Methods

### 2.1. Sourcing of EOs and Bacterial Strains

- EOs: The following six EOs used in this research were provided by Ocean Breeze Personal Care, Casablanca, Morocco: clove (*Syzygium aromaticum*); jasmine *(Jasmin officinal*); pennyroyal (*Mentha pulegium*); rosemary (*Rosmarinus officinalis*); pin (*Pinus sylvestris* var. sylvestris L); and peppermint (*Mentha x piperita*). They were stored at 4°C until utilized.
- Bacterial strains: Four bacterial strains were used in this studying, which three of them are reference bacterial strains including *E. coli* ATCC 25922, *P. aeruginosa* ATCC 27853, and *S. aureus* ATCC 29213, were provided from the collection of the National Institute of Hygiene in Rabat, Morocco. And the fourth one “*S. capitis*” was isolated from contaminated food on specific media as follow: minced meats (poultry and beef) and cheese were left at room temperature for 72 h. Afterward, 10 g of spoiled food was added to 100 mL of sterile distilled water, vortexed until homogenized, then inoculated onto selective media by surface spreading 0.1 mL of the homogenate per Petri dish. The identification of the bacterium was made based on the API STAPH featuring standardized, miniaturized biochemical tests and a specific database.

### 2.2. Disc‐Diffusion Test

The disc‐diffusion method was conducted based on the protocol described by European Committee on Antimicrobial Susceptibility Testing (EUCAST) (2024). First of all, each bacterial suspension was prepared to reach the turbidity standard of 0.5 McFarland Scale. (10^7^–10^8^ CFU/mL). Then, each sterile Petri dish containing sterile Mueller–Hinton Agar (MHA) medium was inoculated with 1 mL of the bacterial suspension to be tested, after 3 min, we removed the excess of the inoculated suspension using plastic pipettes. Afterward, paper disks of 6 mm in diameter were impregnated with 10 *μ*L of the tested EO and deposited in the center of each inoculated Petri plate [5]. On the other hand, the conventional antibiotics (kanamycin [K], chloramphenicol [CLP], and vancomycin [VA]) specific to the following bacteria strain (*E. coli*, *P. aeruginosa*, and *S. aureus*) were used, respectively, as a positive control. To test the growth of the inoculums, we inoculated plates without discs, serving as a negative control. The Petri dishes containing the disc‐diffusion tests and both controls were incubated at 37°C for 16–24 h. Then, the diameters of the inhibition zones were measured by a graduated ruler in millimeters (mm).

According to Wanja et al., the diameter inhibition zone (DIZ) was classified into four categories: A DIZ equal or less than 10 mm is considered inactive. A DIZ ranging from 11 to 18 mm indicate moderate activity, and a DIZ between 19 and 28 mm is considered a strong inhibition, whereas a DIZ equal to or greater than 29 mm suggests a very strong activity [6].

All EOs that showed an inhibition zone inferior to 10 mm, were not examined for the next tests (minimum inhibitory concentration [MIC] and minimum bactericidal concentration [MBC]).

### 2.3. Antibiogram Test

The antibiogram test was conducted based on the recommendation of CLSI (Clinical and Laboratory Standards Institute). The following 12 antibiotics: erythromycin (E); tetracycline (TET); rifampicin (RA); amikacin (AK); VA; imipenem (IPM); colistin (C); amoxicillin (AMX); trimethoprim–sulfamethoxazole (TSX); K; CLP; and nalidixic acid (NA) were tested on the isolated bacterial strain “S. *capitis*”.

### 2.4. MIC

A broth macrodilution method was used to conduct the MIC test, described by EUCAST, 2024. For that, a Mueller–Hinton Broth (MHB) medium was mixed with 0.15% bacteriological agar. The content was poured into a series of eight tubes. The initial tube contained 10 mL and others held 5 mL each, then autoclaved for 15 min at 121°C. Afterward, we added 200 *μ*L into the first tube, then, a series of half dilution was prepared until reaching the concentrations from 20 to 0.156 *μ*L/mL in the final tube.

Subsequently, all these tubes were inoculated with 10 *μ*L of the standardized bacterial suspension. After incubation at 37°C for 16–48 h, the MIC was identified as the lowest concentration preventing the growth of the tested bacterial strains [5].

### 2.5. MBC

In a Petri dish containing MHA medium, 5 *μ*L from each tube that did not show any growth of the bacteria using the MIC test was dropped on the surface at one point. After incubation at 37°C for 16–24 h, the lowest concentration with no evident growth of bacterial strains was considered as MBC [7].

### 2.6. The Ratio of MBC/MIC

To determine the potential of the antimicrobial activity of each EO, the ratio of MBC/MIC was calculated. An EO possesses a bactericidal action if: (MBC/MIC ≈ 1) and a bacteriostatic activity if: (MBC/MIC ≥ 4) [8].

### 2.7. Combined EO With Antibiotic

We tested each antibiotic cited above in combination with the most effective EO against the most resistant bacterial strain in our study (*S. capitis*) by impregnating each antibiotic with 10 *μ*L of *S. aromaticum* EO.

### 2.8. Phytochemical Screening Using GC‐MS (Gas Chromatography‐Mass Spectrometry) Analysis

The phytochemical screening of each tested EO was carried out using the GC‐MS analysis method (HP 6890 series). For this purpose, a single HP DB‐5 capillary column with dimensions of 30 m × 0.25 mm internal diameter and a film thickness of 0.32 *μ*m was used. Nitrogen was utilized as the carrier gas at a flow rate of 1 mL/min.

First of all, each EO was diluted in CHCl_3_ at a ratio of 1:5 v/v, and an injected volume of 0.1 *μ*L was used. The column temperature was programmed to rise from 50°C to 250°C at a rate of 4°C/min, after which it was kept at 250°C for 20 min. The injector temperature was set to 260°C, and a 10:1 split ratio was applied. Fragmentation was achieved by adopting an electronic impact with a field intensity of 70 eV. The identification technique relies on comparing the mass spectrum acquired for each component of the EO with a reference bank of spectra from standard products. The relative proportions of the EO constituents were quantified as percentages derived from normalizing peak areas, with all relative response factors assigned a value of one [9].

### 2.9. In Vitro DPPH (2,2‐Diphenyl‐1‐Picrylhydrazyl) Free Radical Scavenging Assay

DPPH is violet in solution, with an absorption maximum at 517 nm. The antioxidant power of the EOs tested was estimated by comparison with ascorbic acid as a natural antioxidant.

DPPH radical scavenging activity was measured using the protocol described by Blois, where 100 *μ*L of each of the methanolic solutions of the EOs tested at different concentrations are mixed with 1000 *μ*L of DPPH methanolic solution (0.004%). After 30‐min incubation period at laboratory temperature, the absorbance is read at 517 nm. Inhibition of the DPPH by ascorbic acid was also analyzed for comparison.

The percentage of inhibition activity was calculated through the following formula:

%of inhibition=Ac−As÷Ac×100

where Ac is the control reaction absorbance and As is the testing specimen absorbance.

The kinetics of the reactions of EOs and ascorbic acid with DPPH were recorded at each concentration examined. The concentrations of EOs and ascorbic acid, as a function of the inhibited percentages of DPPH, were plotted at the end of the reactions to obtain the EC50 (half maximal effective concentration) index. This parameter is defined as the antioxidant concentration required to reduce the initial DPPH concentration by 50%. The lowest EC50 is translated by the best antioxidant activity.

All tests were carried out with three repetitions [10].

### 2.10. Statistical Analysis

The results were given as the mean ± standard deviation (SD) based on three independent replicates (*n* = 3). The comparison between means was performed by two‐way analysis of variance (ANOVA) and Tukey′s post hoc test using Graphpad prism software (V. 9.5), whereas principal component analysis (PCA) was performed by JMP PRO (V. 16°).

### 2.11. PCA

PCA is a powerful technique in data analysis and machine learning. PCA is widely used for dimension reduction by transforming high‐dimensional data into a lower dimensional space while preserving maximum variance. The objectives of this comprehensive analysis are to elucidate the relationships between the chemical composition of the EOs and their biological activities, identify patterns and clusters among the six EOs based on their overall profiles, and provide insights into the potential applications of these EOs in antimicrobial and antioxidant contexts.

## 3. Results and Discussion

### 3.1. The Phytochemical Screening

The chemical composition of each of the six EOs is given according to the identified compounds and their relative quantities in Table 1.

- *P. sylvestris* var. *sylvestris* L. EO: Twenty‐two compounds were identified, accounting for 87.21% of the total *P. sylvestris* EO composition. The major compounds were terpinen‐4‐ol (24.24%), D‐limonène (13.29%), 3‐carene (7.41%), *α*‐pinene (6.07%), ?‐terpinene (5.66%), *α*‐terpineol (5.17%), bornylacetate (4.25%), *β*‐caryophyllene (3.45%), and *β*‐caryophyllene oxide (3.33%). The chemical composition of *P. sylvestris* EO has been the subject of a number of studies, yet there is a wide variety in the major compounds and their quantities from one study to another. For instance, *α*‐pinene (35.5%), *β*‐pinene (18.6%), *δ*‐3‐carene (15.6%), limonene (11.3%), *β*‐caryophyllene (5.3%), and bornyl acetate (4.5%) were the main component in *P. sylvestris* of Poland [11], whereas Karkok reported 3‐carene (31.55%) and camphene (10.40%) as the major compounds in a study conducted in Turkey. [12]
- *J. officinal* EO: Twenty compounds were identified (94.48% of the total composition of the EO), among which n‐hexyl cinnamaldehyde (23.61%), phenylethyl alcohol (11.85%), benzyl acetate (11.77%), linalool (8.23%), lilial (7.52%), myrcenol (7.16%), and nerolin (5.50%) were as well the predominant compounds. In accordance with our results, some previous studies reported benzyl acetate (11.50%), benzyl benzoate (8.14%), and linalool (2.76%) as being the main compounds [13]. Nonetheless, other study revealed different molecules to be the main volatiles in *J. officinal* EO, such as borneol (37.7%), (R)‐(+)‐limonene (19.2%), benzyl benzoate (10.9%), and 2‐undecanone (4.6%) [14].
- *M. pulegium* EO: The GC‐MS analysis of *M. pulegium* EO in our study has enabled the identification of 19 compounds accounting for 98.5% of the EO composition. The result revealed that the main components were pulegone (73.07%) and menthone (8.81%), followed by piperitenone in a minor amount (2.65%). Several studies reported pulegone and menthone as being the predominant molecules in *M. pulegium* EO; a difference in the major compounds′ percentages depending on the sampling countries was noticed. It has been found pulegone (73.33%) and menthone (35.9%) in Morocco [15] is strongly in agreement with our results. Nonetheless, other studies revealed great variability in the chemical composition of *M. pulegium* EO; they have noticed pulegone and D‐limonene as the main compound [16].
- *S. aromaticum* EO: A total of 97.04% of the chemical composition of *S. aromaticum* EO was identified, represented by six compounds. The major compound was eugenol with an amount of 73.5% followed by *β*‐caryophyllene with 19.22%. Humulene showed an average amount of 3.42%. *α*‐Cubebene, *β*‐caryophyllene oxide, and pentachloroacetone have been detected in *S. aromaticum* EO as a trace. In line with our findings, some previous investigations reported that eugenol (72.66%) and *β*‐caryophyllene (17.41%) are the major components in *S. aromaticum* EO [17]. However, among nine molecules that were identified by El Faqer et al., eugenol (78.67%) and eugenol acetate (11.77%) were the predominant compounds [18].
- *M. piperita* EO: The GC‐MS analysis of *M. piperita* EO revealed the identification of 99.22% of the total composition of the EO. According to the results, the major compounds were menthol (44.67%), menthone (32.84%), 3‐methylcyclohexanol (6.44%), and menthol acetate (5.05%). Aligning with our findings, many other studies detected the predominance of menthol, followed by menthone [40, 41], with slight differences in the percentage and additional compounds like menthofura. However, in a study carried out by Li et al., the dominant compounds were 3,4,5,6,7,8‐hexahydro‐2(1 H)‐naphthalenone, L‐menthol, and pulegone [19].
- *R. officinalis* EO: The GC‐MS analysis of *R. officinalis* EO identified 18 compounds, with major components being eucalyptol (41.82%), pulegone (15.43%), and *β*‐pinene (4.77%). Studies from other countries show eucalyptol as the major constituent followed by other varying elements such as *α*‐pinene (up to 31.3%) from Saudi Arabia [20], camphor (3.3–42.2%), *α* and *β*‐pinene (10.92 and 8.83%) from the USA [21], whereas another study reported different primary compounds like *α*‐pinene (21.5%), bornyl acetate (16.8%), and borneol (10.2%) from Southwest Iran [22].

**Table 1:** The chemical composition of the six tested essential oils.

Compounds	RT	The composition of essential oils (%)					
		*P. sylvestris*	*J. officinal*	*M. pulegium*	*S. aromaticum*	*M. piperita*	*R. officinalis*
Styrene	0.93	—	—	0.05	—	—	—
*α*‐Pinene	1.21	6.07	—	0.21	—	0.16	—
Camphene	1.31	0.42	—	—	—	—	4.40
3‐Methylcyclohexanone	1.39	—	—	0.51	—	—	—
*β*‐Pinene	1.56	—	—	0.22	—	0.17	4.77
3‐Carene	1.59	7.41	—	—	—	—	—
Pentachloroacetone	1.64	—	—	—	0.02	—	—
3‐Octanol	2.05	—	—	0.48	—	0.26	—
2‐Carene	2.09	2.09	—	—	—	—	—
D‐Limonene	2.23	13.29	0.15	1.42	—	2.02	—
?‐Terpinene	2.78	5.66	—	—	—	—	—
*α*‐Terpinolene	3.16	1.24	—	—	—	—	0.55
Dipropylene glycol	3.19	—	0.20	—	—	—	—
Linalool	3.87	—	8.23	—	—	0.16	1.82
Phenylethyl alcohol	4.50	—	11.85	—	—	—	—
Menthofuran	4.52	—	—	0.63	—	—	—
Menthone	4.87	—	—	8.81	—	—	—
Benzyl acetate	5.09	—	11.77	—	—	—	—
Cyclomethicone 5	5.11	—	—	2.45	—	—	—
Terpinen‐4‐ol	5.35	24.24	—	—	—	—	—
Myrcenol	5.57	—	7.16	—	—	—	—
*α*‐Terpineol	5.59	5.17	—	—	—	0.88	2.98
Benzaldehyde, 4‐hydroxy‐3‐methyl‐	5.73	1.27	—	—	—	—	—
p‐Menthane	6.09	0.85	—	—	—	—	—
Pulegone	6.92	—	—	73.07	—	3.55	15.43
Bornylacetate	7.40	4.25	—	—	—	—	—
Piperitenone	7.99	—	—	2.65	—	—	—
*α*‐Tricylene	8.24	—	—	—		—	0.16
*α*‐Longipinene	8.38	0.84	—	—	—	—	—
Car‐3‐en‐5‐one	8.54	0.78	—	0.12	—	—	—
*α*‐Terpineol acetate	8.92	—	1.90	—	—	—	—
*α*‐Cubebene	9.12	—	—	—	0.27	—	—
Cyclomethicone 6	9.14	1.09	0.14	1.38	—	—	—
Longifolene	9.55	2.95	—	—	—	—	—
*α*‐Toluic acid	9.65	—	0.18	—	—	—	—
*β*‐caryophyllene	10.04	3.45	—	—	19.22	—	—
2‐Phenylethyl methoxyacetate	10.09	—	2.17	—	—	—	—
Eugenol	10.17	—	—	—	73.50	—	—
1‐Octen‐3‐ol	10.50	—	—	—	—	—	0.25
3‐Octanone	10.61	—	—	—	—	—	0.08
*α*‐Myrcene	10.77	—	—	—	—	—	1.44
Humulene	10.95	0.60	—	1.09	3.42	—	—
Nerolin	10.97	—	5.50	—	—	—	—
*α*‐Phellandrene	11.20	—	—	—	—	—	0.29
Cyclamal	11.49	—	2.92	—	—	—	—
m‐Cresol	11.66	—	—	—	—	—	0.32
*β*‐Cymene	11.94	—	—	—	—	0.08	—
m‐Xylene	12.07	—	—	—	—	—	0.33
1,8‐Cineole	12.29	—	—	—	—	—	41.82
*δ*‐Cadinene	12.53	0.70	—	—	—	—	—
Cyclomethicone 7	13.00	—	0.34	1.61	—	—	—
Lilial	13.02	—	7.52	—	—	—	—
Phenoxyethyl isobutyrate	13.18	—	2.74	—	—	—	—
1‐Octanol	13.79	—	—	—	—	0.21	—
n‐Hexyl benzoate	14.10	—	1.06	—	—	—	—
*α*‐Terpinene	14.28	—	—	—	—	0.07	—
Anozol	14.71	—	3.33	—	—	—	—
Humulene oxide II	15.06	0.38	—	—	—	—	—
Carveol	16.10	—	—	—	—	0.07	—
n‐Hexyl salicylate	16.13	—	3.34	—	—	—	—
Isopulegol	16.43					1.80	1.13
Menthone	16.77	—	—	—	—	32.84	—
Citronellal	17.17	—	—	—	—	—	4.31
3‐Methylcyclohexanol	17.19	—	—	—	—	6.44	—
Menthol	17.66	—	—	—	—	44.67	—
n‐Hexyl cinnamaldehyde	17.90	—	23.61	—	—	—	—
Galaxolide	19.04	—	0.32	—	—	—	—
D‐Carvone	19.86	—	—	—	—	0.07	—
Ketone musk	20.73	—	0.39	—	—	—	—
Isobornylacetate	21.25	—	—	—	—	—	0.71
Benzeneacetic acid	21.98	—	—	0.27	—	—	—
Menthol acetate	22.02	—	—	—	—	5.05	—
Arachidonic acid	22.79	—	—	0.22	—	—	—
3‐(2‐Phenylethyl) benzonitrile	23.44	—	—	1.31	—	—	—
*β*‐Caryophyllene oxide	23.67	3.33	—	1.00	0.61	—	—
3‐Benzyloxy‐1,2‐diacetyl‐1,2‐propanediol	24.44	1.16	—	—	—	—	—
1‐Butylpyrrole	24.53	—	—	—	—	0.30	—
Cumaldehyde	25.65	—	—	—	—	—	4.67
Nerolidol	30.56	—	—	—	—	0.10	—
Total		87.21%	94.82%	98.5%	97.04%	99.22%	99.16%

Abbreviation: RT, retention time.

The differences in the chemical compounds might be due to drying methods, screening test, collecting periods, geographical sites, soil composition, climate, genotypes, and chemotypes [23].

### 3.2. The Antioxidant Activity

In light of the exploration of bioactive compounds derived from plants, the current research investigated the potential of six distinct EOs for their antioxidant activity. The DPPH assay results demonstrate varying antioxidant activities among the six tested EOs, as indicated by their EC50 values (Table 2). *S. aromaticum* EO exhibits the strongest antioxidant potential with the lowest EC50 value (0.019 mg/mL), compared with ascorbic acid, a reference compound, making it the most effective at scavenging free radicals, which could be justified by its chemical composition, rich in eugenol (73.50%), as well as through the synergistic interactions between eugenol and the other minor compounds found in this EO [24].

**Table 2:** DPPH *(*2,2‐diphenyl‐1‐picrylhydrazyl) activity of the six EOs (EC50).

EOs	EC50 (mg/mL)
*P. sylvestris*	2.380 ± 0.270
*J. officinal*	1.150 ± 0.350
*S. aromaticum*	0.019 ± 0.002
*M. piperita*	25.520 ± 0.050
*R. officinalis*	12.370 ± 0.060
*M. pulegium*	39.910 ± 0.030
Ascorbic acid	0.063 ± 0.000

*J. officinal* and *P. sylvestris* also show relatively strong antioxidant activities, with EC50 values of 1.15 and 2.38 mg/mL, respectively. In contrast, *M. piperita*, *R. officinalis*, and *M. pulegium* EOs display weaker antioxidant effects, with EC50 values ranging from 12.37 to 39.91 mg/mL. Notably, *M. pulegium* has the weakest antioxidant activity. This result is close to that reported by Alphonse et al., who found (0.02317 mg/mL) for *S. aromaticum* collected in Cameroon [25] but higher than the values found by Saeed and Shahwar (0.0045 mg/mL) in Pakistan [26].

### 3.3. The Antimicrobial Activity

- *Disc-diffusion test:*

Table 3 summarizes the diameters of inhibition zones of the six EOs and the antibiotics against three tested reference bacterial strains. The values ranged from 0 to 34.50 ± 5.50 mm. The largest diameter was observed against *E. coli* when treated with *S. aromaticum* EO (34.50 ± 5.50 mm), indicating that this EO exhibited very strong antimicrobial activity against this bacterium, followed by *S. aureus* (22.33 ± 4.62 mm), whereas *M. pulegium* EO was more effective against *P. aeruginosa* (20.00 ± 5.00 mm). Our results were higher than those found by Afanyibo et al., who demonstrated an inhibition zone varying from 12.00 to 18.00 mm against *E. coli* ATCC 25922 when treated with the extract of *S. aromaticum*. However, the same authors found a quite similar inhibition zone against *P. aeruginosa* ATCC 27853 (13.00–19.00 mm) and *S. aureus* ATCC 29213 (19.00–22.00 mm) [27].

**Table 3:** Inhibition Zone of the EOs and antibiotics against reference bacterial strains.

Category	Tested agent	Bacterial strains	Inhibition zone (mm)	Inhibition potential
E.Os	*S. aromaticum*	*E.coli*	34.50 ± 5.50b ^b^	Very strong activity
		*P. aeruginosa*	19.33 ± 2.52 ^a^	Strong activity
		*S. aureus*	22.33 ± 4.62 ^a^	Strong activity
	*M. pulegium*	*E. coli*	12.67 ± 1.53^a^	Moderate activity
		*P. aeruginosa*	20.00 ± 5.00^b^	Strong activity
		*S. aureus*	19.67 ± 4.62 ^b^	Strong activity
	*M. piperita*	*E. coli*	19.00 ± 1.00 ^b^	Strong activity
		*P. aeruginosa*	12.00 ± 1.0 ^a^	Moderate activity
		*S. aureus*	13.67 ± 2.31 ^a^	Moderate activity
	*J. officinal*	*E. coli*	9.67 ± 0.58 ^a^	Inactive
		*P. aeruginosa*	11.67 ± 2.08 ^a^	Moderate activity
		*S. aureus*	10.67 ± 0.58 ^a^	Moderate activity
	*R. officinalis*	*E. coli*	10.00 ± 0.00 ^a^	Inactive
		*P. aeruginosa*	14.33 ± 0.58^a^	Moderate activity
		*S. aureus*	12.33 ±1.53 ^a^	Moderate activity
	*P. sylvestris*	*E. coli*	0.00 ± 0.00^a^	Inactive
		*P. aeruginosa*	11.50 ± 0.50 ^b^	Moderate activity
		*S. aureus*	13.50 ± 1.00 ^b^	Moderate activity
Antibiotics	Kanamycin	*E. coli*	18.00 ± 0.00	Moderate activity
	Chloramphenicol	*P. aeruginosa*	20.00 ± 0.00	Strong activity
	Vancomycin	*S. aureus*	15.00 ± 0.00	Moderate activit

*Note:* Within every EO, means with different superscript letters (a and b) were significantly different at the level of *p* < 0.05.

A two‐way ANOVA revealed a significant interaction effect (*p* < 0.0001) between EO type and bacterial strain. This key finding indicates that the effectiveness of a given EO is not uniform across all bacterial strains, and conversely, the susceptibility of a given strain varies depending on the specific oil used. Furthermore, significant main effects were observed for both the type of EO (*p* < 0.0001) and the bacterial strain (*p* < 0.0001). The significant main effect of EO type suggests inherent differences in the overall antibacterial potency among the six oils tested, irrespective of the bacterial strain. Similarly, the significant main effect of bacterial strain indicates inherent differences in susceptibility among the three strains, irrespective of the oil applied.

The Tukey′s post hoc test reveals some information about the antibacterial effect of each of the six EOs on the three strains studied. Notably, *J. officinal* and *R. officinalis* EOs exhibited identical efficacy across all three reference bacterial strains tested. In contrast, within each of the remaining four EOs (*S. aromaticum*, *M. pulegium*, *P. sylvestris*, and *M. piperita*), similar antibacterial activity was demonstrated against *P. aeruginosa* and *S. aureus* while exhibiting a distinct effect on *E. coli.* In addition to the largest inhibition zones shown by *S. aromaticum*, this EO was more effective than two of the three tested antibiotics. It showed an inhibition zone two times higher than that found by K against *E. coli*, 33% higher than that registered by VA on *S. aureus*, and quite similar to that illustrated by CLP against *P. aeruginosa*. (Table 3).

In our research, both (*P. aeruginosa* and *S. aureus*) were also strongly inhibited by *M. pulegium* EO, and it showed a similar inhibition zone to that found by the antibiotic CLP on *P. aeruginosa* and higher to the VA against *S. aureus*. (Table 3). Contrasting with the findings reported by Ez‐Zriouli et al., who illustrated an inactive effect of *M. pulegium* on *P. aeruginosa* (7.00 mm) and a moderate impact against *S. aureus* (17.33 mm) [28]. However, *E. coli* in the present research was noticed to be the most resistant bacterial strain, because no inhibition zone was observed when treated with *J. officinal*, *R. officinalis*, or *P. sylvestris* EOs. Consequently, these three EOs were not evaluated for the MIC and MBC tests against *E.coli*.

In general, according to the presented results of the inhibition zone, *M. piperita* EO was recorded to be efficient against *E. coli*, whereas *M. pulegium* was effective on *P. aeruginosa* and *S. aureus*. Moreover, *S. aromaticum* was illustrated as the most efficient EO against all tested bacterial strains. For that, these three EOs were tested on the isolated *S. capitis*, which showed multiresistance to all‐tested antibiotics (Table 4).

**Table 4:** The DIZ of three EOs and the antibiotics against *S. capitis*.

Category	Tested agent	Inhibition Zone (mm)	Inhibition Potential
EOs	*S. aromaticum*	20,00 ± 0,00	Strong activity
	*M. pulegium*	15,00 ± 0,00	Moderate activity
	*M. piperita*	14,00 ± 0,00	Moderate activity
Antibiotics	IPM	0 ± 0,00	Inactive
	TET	0 ± 0,00	Inactive
	R	0 ± 0,00	Inactive
	TSX	0 ± 0,00	Inactive
	AMX	0 ± 0,00	Inactive
	E	0 ± 0,00	Inactive
	C	0 ± 0,00	Inactive
	K	0 ± 0,00	Inactive
	VA	0 ± 0,00	Inactive
	CLP	0 ± 0,00	Inactive
	AK	0 ± 0,00	Inactive
	NA	0 ± 0,00	Inactive

*Note:* Inhibition Zone (DIZ); Imipenem (IPM); Tetracycline (TET); Rifampicin (R); Trimethoprim‐Sulfamethoxazole (TSX); Amoxicillin (AMX); Erythromycin (E); Colistin (C); Kanamycin (K); Vancomycin (VA); Chloramphenicol (CLP); Amikacin (AK); Nalidixic acid (NA).

The results showed a strong inhibition activity on *S. capitis* while treated with *S. aromaticum* EO (20 mm). A moderate effect was recorded while using *M. pulegium* (15 mm) or *M. piperita* (14 mm). However, *S. capitis* demonstrated significant multiresistance to all 12 tested antibiotics (Table 4); these results are confirmed by many previous studies who elucidated the multiresistance of *S. capitis* to various antibiotics [29, 30].

- Determination of the MIC, MBC, and the ratio MBC/MIC

The lowest MIC of the isolated strain (*S. capitis*) was witnessed in *S. aromaticum* at 0.63 *μ*L/mL, followed by 2.5 *μ*L/mL of *M. piperita,* and then 3.33 *μ*L/mL of *M. pulegium*. However, only *S. aromaticum* could be considered as the effective EO against this bacterium, which illustrated the lowest MBC of 1.67 *μ*L/mL, conferring by the way a bactericidal effect on *S. capitis*, whereas *M. piperita* and *M. pulegium* failed to demonstrate bactericidal activity against *S. capitis*, even at the highest concentration tested (20 *μ*L/mL) (Table 5).

**Table 5:** The MIC, MBC, and the ratio (MBC/MCI) of the three EOs against *S. capitis.*

EOs	Bacterial strains	MIC	MBC	Ratio MBC/MIC	Antimicrobial potential
*S. aromaticum*	*S. capitis*	0.63 ± 0.00	1.67 ± 0.00	2.67	Bactericidal
*M. pulegium*		3.33 ± 1.44	> 20	> 20	Bacteriostatic
*M. piperita*		2.50 ± 0.00	20.00 ± 0.00	8.00	Bacteriostatic

Abbreviations: EOs, essential oils; MIC, minimum inhibitory concentration; MBC, minimum bactericidal concentration; *S. capitis*, *Staphylococcus capitis.*

In general, the most efficient EO in our research was attributed to *S. aromaticum* EO. Consequently, its antimicrobial activity was further evaluated in combination with antibiotics against the isolated bacterium *S. capitis.* The corresponding results are discussed in the next section. On the other hand, the minimum inhibitory and MBCs of the six studied EOs against the reference strains ranged, respectively, from 0.63 to > 20.00 *μ*L/mL and 1.00 to > 20.00 *μ*L/mL (Table 6).

**Table 6:** The MIC, MBC, and the ratio (MBC/MCI) of the six EOs on the three reference strains.

EOs	Bacterial strains	MIC	MBC	Ratio MBC/MIC	Antimicrobial potential
*S. aromaticum*	*E. coli*	2.08 ± 0.72	2.08 ± 0.72	1.00	Bactericidal
	*P. aeruginosa*	1.46 ± 0.95	1.67 ± 0.72	1.14	Bactericidal
	*S. aureus*	1.25 ± 0.00	2.08 ± 0.72	1.67	Bactericidal
*M. pulegium*	*E. coli*	7.50 ± 4.33	7.50 ± 4.33	1.00	Bactericidal
	*P. aeruginosa*	9.17 ± 9.46	10.00 ± 8.66	1.09	Bactericidal
	*S. aureus*	4.17 ± 1.44	8.33 ± 2.89	2.00	Bactericidal
*M. piperita*	*E. coli*	4.17 ± 1.44	4.17 ± 1.44	1.00	Bactericidal
	*P. aeruginosa*	4.17 ± 1.44	5.00 ± 0.00	1.20	Bactericidal
	*S. aureus*	8.33 ± 2.89	8.33 ± 2.89	1.00	Bactericidal
*J. officinal*	*E. coli*	10.00 ± 0.00	10.00 ± 0.00	1.00	Bactericidal
	*P. aeruginosa*	2.50 ± 0.00	20.00 ± 0.00	8.00	Bacteriostatic
	*S. aureus*	2.50 ± 0.00	5.00 ± 0.00	2.00	Bactericidal
*R. officinalis*	*E. coli*	8.33 ± 2.89	11.67 ± 7.64	1.40	Bactericidal
	*P. aeruginosa*	10.00 ± 0.00	10.00 ± 0.00	1.00	Bactericidal
	*S. aureus*	5.00 ± 0.00	1.00 ± 0.00	1.00	Bactericidal
*P. sylvestris*	*E. coli*	DIS	DIS	DIS	DIS
	*P. aeruginosa*	> 20	DIS	DIS	DIS
	*S. aureus*	> 20	DIS	DIS	DIS

Abbreviations: DIS, discarded; EOs, essential oils; MIC, minimum inhibitory concentration; MBC, minimum bactericidal concentration; *S. capitis, Staphylococcus capitis.*

The antimicrobial susceptibility observed in our study through the MIC test indicates a greater impact on Gram‐positive reference bacteria (*S. aureus*), except for *M. piperita,* where this bacterium was more resistant and needed a double concentration (8.00 *μ*L/mL) than that required for the Gram‐negative reference bacteria (*E. coli* and *P. aeruginosa*) (4.00 *μ*L/mL). Normally, the resilience of Gram‐negative bacteria to the majority of EOs is often due to their hydrophilic outer membrane. This characteristic prevents the entry of hydrophobic compounds into the membrane of the intended cell [30].

On the other hand, *P. sylvestris* EO did not inhibit the growth of all the studied bacterial strains even with the highest concentration (20 *μ*L/mL), therefore, the MBC was not calculated. Our results odds with those found by Loizzo et al., who demonstrated an antimicrobial susceptibility of pine EO growing in Lebanon with an MIC ranging from 6.00 to 8.00 *μ*L/mL against *S. aureus* (ATCC 25923) and an MIC between 8.00 and 9.00 *μ*L/mL on *E. coli* [31]. However, the same study found that *P. aeruginosa* (ATCC 27853) was not inhibited even with the maximum concentration tested > 10 *μ*L/mL [31]. The variation in the results might be explained by the differences in the plant species; therefore, the divergent in the chemical composition resulting in a disparity in the biological activities. In the present study, the major compound found in *P. sylvestris* EO was terpinen‐4‐ol (24,24%). Conversely, Loizzo et al. revealed *α*‐pinene (40.70%) as the main compound [31]. This element was commonly reported by its antibacterial properties when tested against some microorganisms [32].

Moreover, apart from *P. sylvestris*, the remaining five EOs demonstrated quite similar MIC and MBC concentrations indicating a bactericidal effect on all tested reference strains, whereas, *J. officinal* EO showed a much higher MBC of 20 *μ*L/mL on *P. aeruginosa* indicating by the way the only bacteriostatic effect of this EO against this bacterium.

- *Combined EO with antibiotic test*

According to Table 7, and regarding the combined effect of the antibiotics and *S. aromaticum* EO, we noticed that IPM showed the highest inhibition zone of 40 mm, followed by TET with a DIZ = 32 mm, and then R as well as TSX with a similar DIZ = 29 mm. Moreover, AMX, E, C, K, VA, CLP, and AK illustrated a strong antimicrobial activity ranging from 19 to 24 mm. However, the combination with the antibiotic NA yielded moderate activity with a DIZ of 17 mm (Table 7).

**Table 7:** The DIZ of the combined effect of antibiotics with *S. aromaticum* EO on *S. capitis.*

Antibiotics	DIZ (mm)	Inhibition potential
IPM	40.00 ± 0.00	Very strong
TET	32.00 ± 0.00	Very strong
R	29.00 ± 0.00	Very strong
TSX	29.00 ± 0.00	Very strong
AMX	24.00 ± 0.00	Strong
E	23.00 ± 0.00	Strong
C	22.00 ± 0.00	Strong
K	21.00 ± 0.00	Strong
VA	20.00 ± 0.00	Strong
CLP	20.00 ± 0.00	Strong
AK	19.00 ± 0.00	Strong
NA	17.00 ± 0.00	Moderate

Abbreviations: AK, amikacin; AMX, amoxicillin; C, colistin; CLP, chloramphenicol; DIZ, diameter inhibition zone; E, erythromycin; IPM, imipenem; K, kanamycin; NA, nalidixic acid; R, rifampicin; TET, tetracycline; TSX, trimethoprim–sulfamethoxazole; VA, vancomycin.

In general, the DIZ of *S. aromaticum* alone showed strong inhibition with a value of 20 mm (Table 4), the combined effect of the antibiotics along with this EO appears to have performed a synergetic effect and enhanced the antimicrobial effect of eight antibiotics including (IPM; TET; R; TSX; AMX; E; C; and K). However, the inhibition zone of the same EO combined with VA or CLP antibiotics remained 20 mm, whereas the DIZ of *S. aromaticum* with AK and NA declined, illuminating an antagonist effect.

### 3.4. PCA

The PCA was applied to assess the relationships between the major chemical compounds, antioxidant, and antibacterial activities of the EOs. Based on Kaiser′s criterion (1960), in a normalized PCA, components with eigenvalues greater than 1 should be retained [33]. Collectively, these five components accounted for the entirety of the observed variance (100%). Notably, the first two principal components alone explained 59.78% of the total variability, indicating a significant concentration of pertinent information within these dimensions (Table 8).

**Table 8:** Eigenvalues and explained variance for principal components.

Number	Eigenvalue	Percentage	Cum percentage
1	5.604858	37.366	37.366
2	3.362381	22.416	59.782
3	2.586689	17.245	77.026
4	2.112450	14.083	91.109
5	1.333622	8.891	100.000

**Figure 1:**
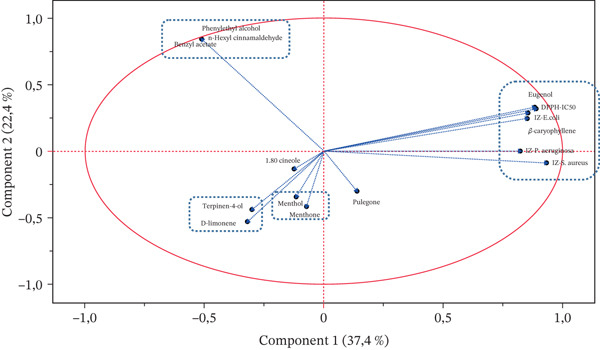
Loading plot of the major chemical compounds, antioxidant activity, and antibacterial activity on the first two principal components.

The loading plot analysis reveals crucial insights into the relationships between chemical constituents and biological activities of the studied EOs (Figure 1). The first principal component is strongly correlated with antibacterial and antioxidant activities, along with the major compounds eugenol and *β*‐caryophyllene, suggesting that oils rich in these compounds may exhibit enhanced biological properties. Along the second component, phenylethyl alcohol, benzyl acetate, and n‐hexyl cinnamaldehyde show robust positive correlations, indicating their frequent co‐occurrence in the analyzed oils.

**Figure 2:**
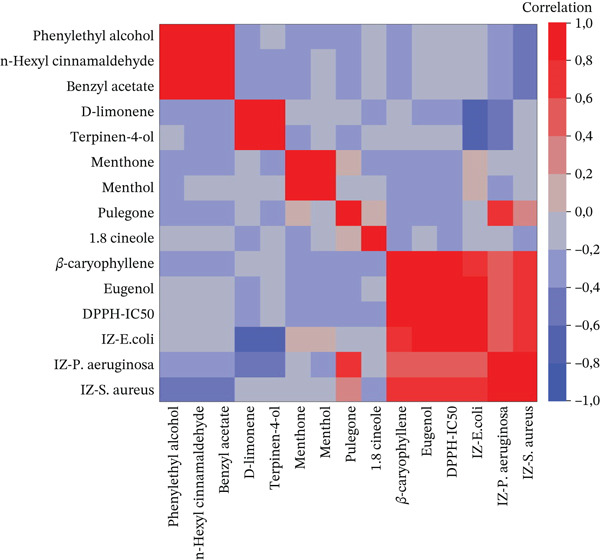
Heatmap of correlations between chemical major compounds of the six studied EOs and antibacterial and antioxidant activities.

Additionally, strong positive correlations are noted between menthol and menthone, as well as between terpinen‐4‐ol and D‐limonene, hinting at potential biosynthetic relationships or common sources for these compound pairs. Furthermore, similar correlations were also confirmed by the heat map graph (Figure 2), indicated by (*r* ≈ 1, *p* < 0.05), suggesting a robust and nonrandom association.

**Figure 3:**
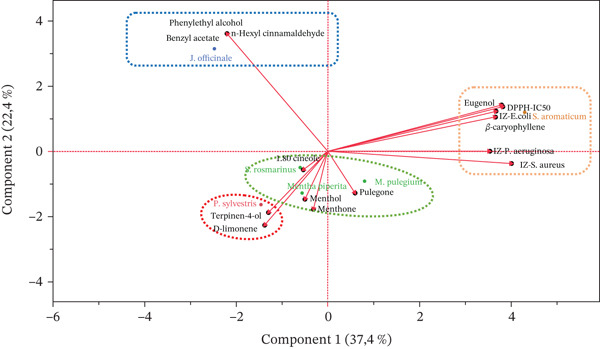
Biplot of physicochemical, antioxidant, and bacteriological variables and fractions of six essential oils on the first two principal components.

In addition, based on the Biplot graph (Figure 3), the PCA analysis revealed four distinct groups among the six analyzed EOs:

- (Group 1) *S. aromaticum*, rich in eugenol and *β*‐caryophyllene, exhibiting the highest antibacterial and antioxidant activities; our findings align with previous studies that have demonstrated the potent antimicrobial and antioxidant effects of eugenol and *β*‐caryophyllene [34].
- (Group 2) (include *M. pulegium*, M. piperita, and *R. officinalis*) characterized by pulegone, menthol, and 1,8‐cineole, respectively, showing moderate bioactivity; suggests a common biochemical basis for their therapeutic properties. The moderate activity of these oils, despite their rich terpene content, raises questions about potential antagonistic interactions or the specific roles of these compounds in biological systems [23].
- (Group 3) *P. sylvestris*, marked by D‐limonene and terpinen‐4‐ol;
- (Group 4) *J. officinal*, distinguished by phenylethyl alcohol, benzyl acetate, and n‐hexyl cinnamaldehyde. The latter two groups (3 and 4) displayed weak to very weak antibacterial and antioxidant activities, challenging by the way, the assumption that all EOs possess strong antimicrobial properties [35].

This classification highlights the diversity of chemical profiles among EOs and their corresponding biological activities, underscoring the relationship between specific chemical constituents and observed bioactivities.

This comprehensive analysis provides a foundation for further investigation into causal relationships between compounds and biological activities, as well as exploration of underlying mechanisms responsible for the observed correlations, thereby advancing our understanding in fields such as natural product chemistry, pharmacognosy, and antimicrobial research.

## 4. Conclusion

In summary, our findings revealed significant antimicrobial activity of all EOs, except for *P. sylvestris* which failed to inhibit the growth of all tested reference bacteria. Although all EOs demonstrated bactericidal effect against the studied bacterial strains, *J. officinal* EO displayed bacteriostatic activity only against *P. aeruginosa.* Moreover, we investigated the antimicrobial activity of *S. aromaticum*, *M. piperita*, and *M. pulegium* on an isolated multiresistant bacterium *S. capitis*. As per our knowledge, no prior studies examined these specific interactions. Thus, our research is aimed at filling this gap by investigating their potential antimicrobial effects on *S. capitis.*

Notably, *S. aromaticum* was the most effective EO due to its high antioxidant activity (EC50 = 0.019 mg/mL) and antimicrobial potential against all tested bacterial strains. Furthermore, the combined effect of this EO with each tested antibiotic enhanced the antimicrobial activity against *S. capitis*, highlighting the synergistic potential of this combination. In addition, the phytochemical screening of each EO revealed the presence of abundant components known to possess antimicrobial properties. The multivariate analysis, combining PCA and correlation studies, provided valuable insights into the complex relationships between chemical composition and biological activities of the six EOs examined. Our findings highlighted the significance of specific compounds in determining bioactivity. Strong positive correlations (*r* ≈ 1, *p* < 0.05) between major compounds (notably eugenol and *β*‐caryophyllene) and antibacterial and antioxidant activities were confirmed through statistical analysis and visualized via a heatmap.

Our findings suggested promising avenues for the development of novel antimicrobial agents, including the synergetic action of antibiotics with *S. aromaticum* EOs, an original way that proves to be much more effective than using either the conventional antibiotics alone. These results highlight the importance of further research to elucidate the specific mechanisms underlying the antimicrobial activity of these EOs and their individual components.

## Funding

No funding was received for this manuscript.

## Conflicts of Interest

The authors declare no conflicts of interest.

## Data Availability

The data used in this study are available from the corresponding author on reasonable request.
